# Structural and geomorphological study of Bradost, Chinara, Shireen and Sare Musa anticlines, Iraqi Kurdistan Region

**DOI:** 10.1016/j.heliyon.2023.e18375

**Published:** 2023-07-16

**Authors:** Varoujan K. Sissakian, Ala A. Gahfur, Nadhir Al-Ansari, Hassan O. Omer, Hawkar A. Abdulhaq

**Affiliations:** a Department of Petroleum Engineering, Komar University of Science and Technology, Sulaymaniyah, KRG, Iraq; b Department of Geology, University of Regina, Canada; c Department of Civil, Environmental and Natural Resources Engineering, Lulea University of Technology, Sweden; d Deventer, the Netherlands; e Szeged University, Hungary

**Keywords:** Lateral growth, Water gap, Wind gap, Thrust faults, Alluvial fans, Landslides

## Abstract

The Bradost and Chinara mountains are two well-known geomorphic features in the Iraqi Kurdistan Region (IKR), forming two anticlines, besides Shireen and Sare Musa anticlines, which are located north of the Bradost anticline, all four anticlines trend NW – SE. The four anticlines are dissected by the Greater Zab River that swings along its course within the anticlines due to tens of very old landslides and/or plunges. The four studied anticlines are dissected by different thrust faults, which extend for a few kilometers. The thrust faults trend NW – SE; however, locally they deflect from the main trend. The Lower Jurassic rocks are the oldest exposed rocks in the studied area, whereas the rocks of the Bekhme Formation form the carapace of the Bradost and Chinara anticlines. Different structural and geomorphological features were interpreted from satellite images and those which are accessible were checked in the field, all of them indicate the four anticlines exhibit lateral growth. We have measured different aspects to elucidate the type of folds. The four anticlines are Detachment folds, with shallow decollement, which ranges in depth between (100–250) m.

## 1 Introduction

The developed anticlines in KRI are due to the active tectonics in the Zagros Fold–Thrust Belt (ZFTB) caused by the convergent collision between the Arabian and Eurasian plates, which started since Upper Cretaceous [[1], [2], [3], [4], [5], [6], [7], [8]]. The IKR occupies the northeastern part of the Arabian Plate; accordingly, has received, and still receiving forces due to the collision of the Arabian and Eurasian plates.

Apart from the Chinara anticline, the other three anticlines have very anomalous shapes, which fairly resemble the normal anticlinal shape, the Chinara anticline; however, also exhibit anomalous shapes but are not extensive as the other three anticlines. The anomalous shapes are attributed to: 1) intensive thrusting of an anticline over an adjacent anticline leading to the disappearance of the syncline in between them, or even one limb over the other leading to the disappearance of the axial part of the anticline, 2) presence of rock with various competencies, 3) en-echelon plunging, 4) extensive erosional and karst forms, 5) development of different valley shapes, 6) development of very deep water and wind gaps, 7) development of steep and high ridges, and 8) different mass movement phenomena along the limbs of the anticlines. According to Burbank and Anderson [9]; Blanc et al. [2], and Fossen [10] some anticlines can exhibit anomalous structural shapes. The Bradost, Shireen, and Sare Musa anticlines have strange structural shape, this is attributed to the aforementioned reasons. Chinara anticline; however, also exhibit an anomalous shape but is not severe like the other three anticlines.

**Fig. 1:**
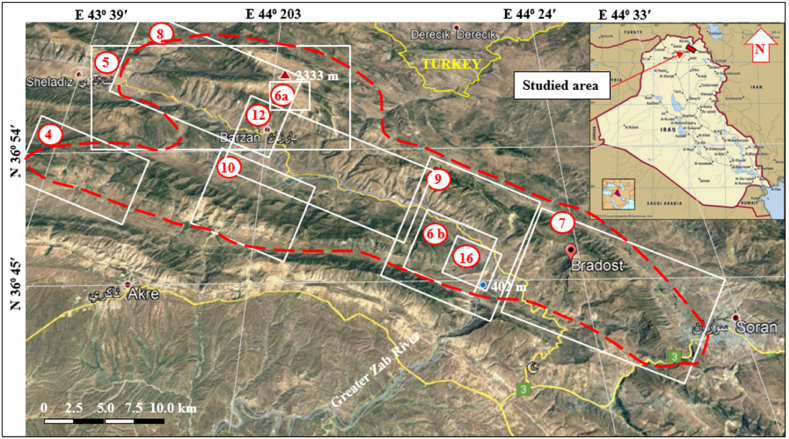
Satellite image of the locations of the four studied anticlines. The studied area is limited approximately by the dashed red color. The triangles with numbers are the locations of the presented figures in the text.

The studied area is located in the central northern part of the Iraqi Kurdistan Region (Fig. 1). The studied area is about 1650 km^2^, and it can be reached via Erbil and Duhok cities.

This study aims to deduce the structural and geomorphological status of the Bradost, Chinara, Shireen, and Sare Musa anticlines. Moreover, to update the structural forms of the concerned anticlines.

## 2 Materials and methods

We have interpreted satellite images, used geological maps [11], and reviewed different published articles to perform the current study and fulfill its aims. Interpreted images, locally show some coverage area to an adjacent image; to present more detailed interpreted data.

We also have added some uncommon symbols directly on some images to clarify the used symbols; like karstified area, old landslide, hanging syncline … etc. Moreover, we have conducted field visits to the accessible parts to check the interpreted data. The studied area is characterized by a very steep and rugged topography, which makes a large obstacle for field checks, besides the presence of thousands of mines, which were haphazardly distributed during many past decades and form a true challenge for fieldwork.

**Fig. 2:**
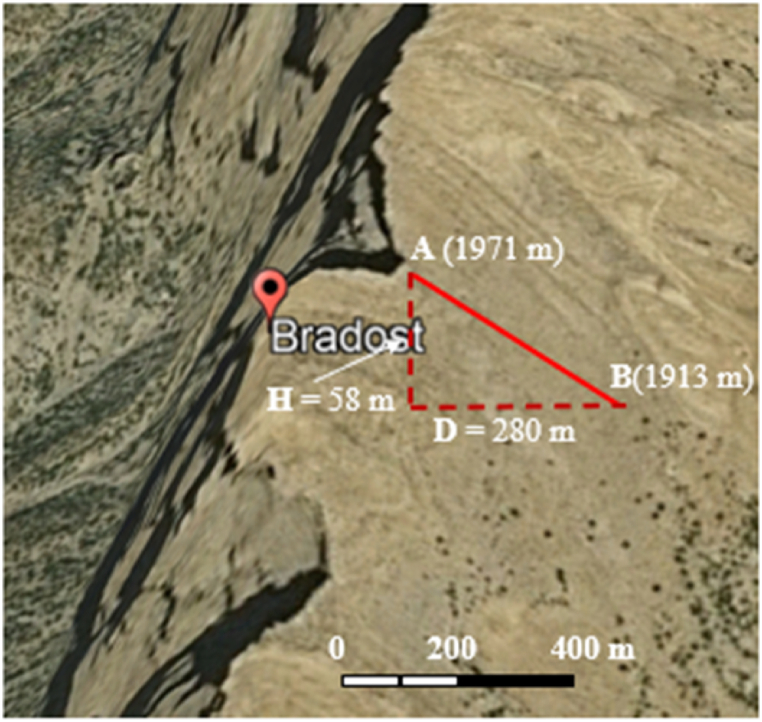
Satellite image demonstrating the procedure of dip calculation.

From the reviewed published articles and books, we have adopted the opinions of different researchers when we recognized significant structural and geomorphological forms and features based on [6,10,[12], [13], [14], [15], [16], [17], [18], [19], [20], [21], [22], [23],], We have calculated dip amounts along both plunges of the four anticlines and the average amount of the dip on both limbs to indicate the axial plane (Table 1) using satellite images. We have selected very clear bedding planes (Fig. 2); after zooming the satellite images to be sure that the measurements are along the same bedding plane and have checked and corrected dip amounts to some of the accessible locations. The average dip amounts of the plunges of each anticline were used to indicate the shape of the fold based on Fleuty [13].

**Table 1:** Calculated geomorphological indices along the Bradost, Chinara, Shireen and Sare Musa anticlines.

Anticline	FS (km)	H. L (km)	L (km)	W (km)	W/2 (km)	S (km)	AR	IFS	Smf	Axial Plane (⁰)	Plunge (⁰)		
											NW	SE	Av.
**Chinara**	59.9	53.1	52.9	5.84	2.92	3.65	9.09	1.25	1.09	33 SW	24	20	22
**Bradost**	54.2	49.8	47.8	9.39	4.69	3.12	5.09	0.67	1.11	40 NE	26	10	18
**Shireen**	41.9	37.7	35.7	5.46	2.73	3.95	6.54	1.45	1.04	41 NE	20	12	16
**Sare Musa**	39.4	34.8	32.2	3.24	1.12	0.91	9.94	0.81	1.30	43 NE	21	55	38

The dip amount was calculated by equation (1):

(1) tan Ø = H/D

where: H is the height difference between points A and B (Fig. 2), D is the distance between A – B, and Ø is the dip angle.

The IFS is calculated by Ref. [24] equation (2):

(2) IFS=S/ (W/2)

where: S is the width of the forelimb, and W is the width of the fold.

**Fig. 3:**
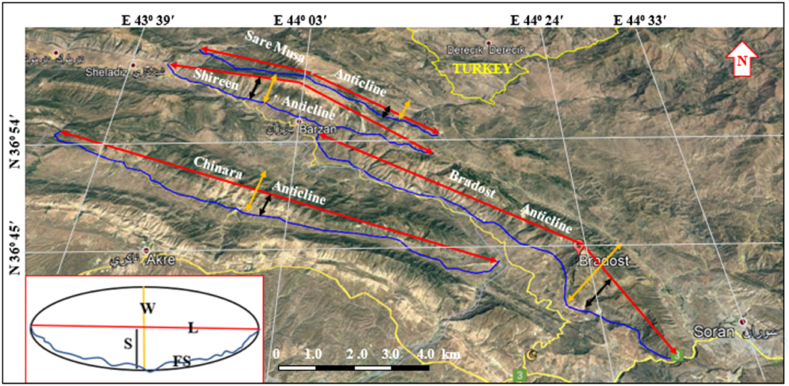
Satellite image showing the calculated parameter.

The AR is calculated by Ref. [23] (Fig. 3) equation (3):

(3) AR = L/W

where: L is the length of the fold, W is the width of the fold.

The Smf is calculated by Ref. [25] equation (4):

(4) Smf = Lmf / Ls

where: Lmf is the length of the mountain front (FS) along the foot of the mountain at the pronounced break in slope, and Ls is the straight-line length of the mountain front (L), and H.L. is the hinge length (Table 1). Table (1) includes the acquired data.

We have presented 11 interpreted satellite images showing different structural and geomorphological forms and/or units; the majority of them were not presented on the published geological maps. This is attributed mainly due to the scale limitations of the published maps. On some images, we have added a small legend to explain the used symbols, other symbols are explained in the title of the image. When there is an overlap between two presented images, then the interpreted structural and/or geomorphological forms are presented only on one of the two interpreted images; to avoid duplication.

## 3 Geology of the studied anticlines

The geology of the studied area is briefed hereinafter based on [[26], [27], [28]].

### 3.1 Geomorphology

**Fig. 4:**
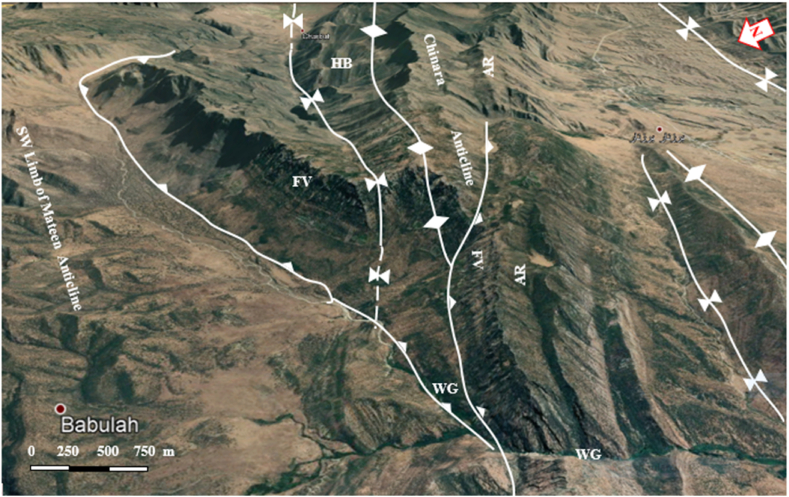
Satellite image of the Chinara anticline (NW plunge), note the Hogbacks (HB), Anticlinal ridges (AR), Water gaps (WG), and Fork-shaped valleys (FV).

**Fig. 5:**
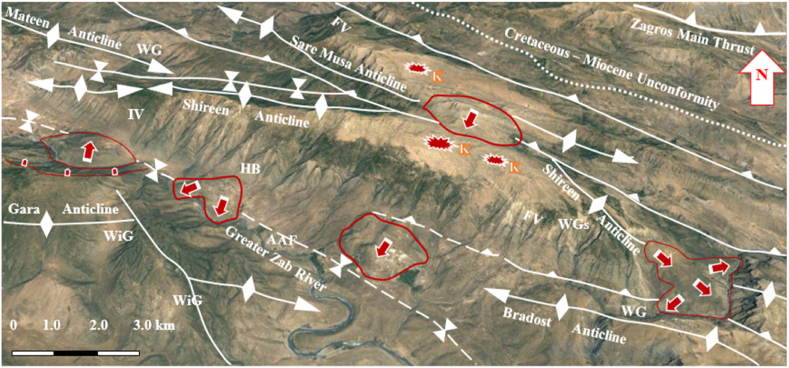
Satellite image of the Shireen and Sare Musa anticlines. Note the Water (WG) and Wind gaps (WiG), Wine glass (WGs), Inclined valleys (IV), Fork-shaped valleys (FV), Landslides (red arrows), and Karstified areas (K). Figure (1) shows the location of the image.

**Fig. 6:**
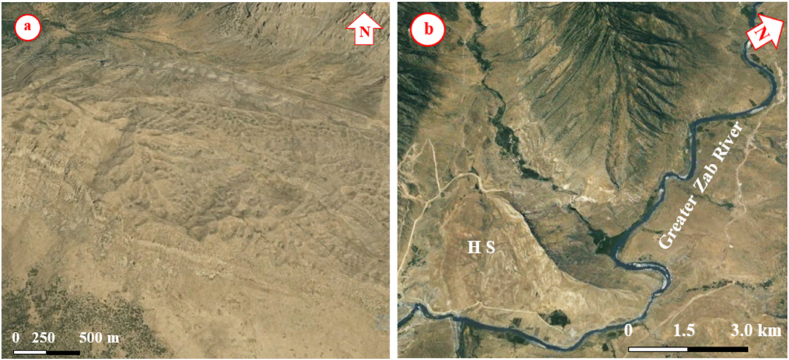
Satellite image, a) Intensively karstified area on top of Shireen anticline, b) typical Radial valleys on the SE plunge of the Chinara anticline, and a hanging syncline (HS).

**Fig. 7:**
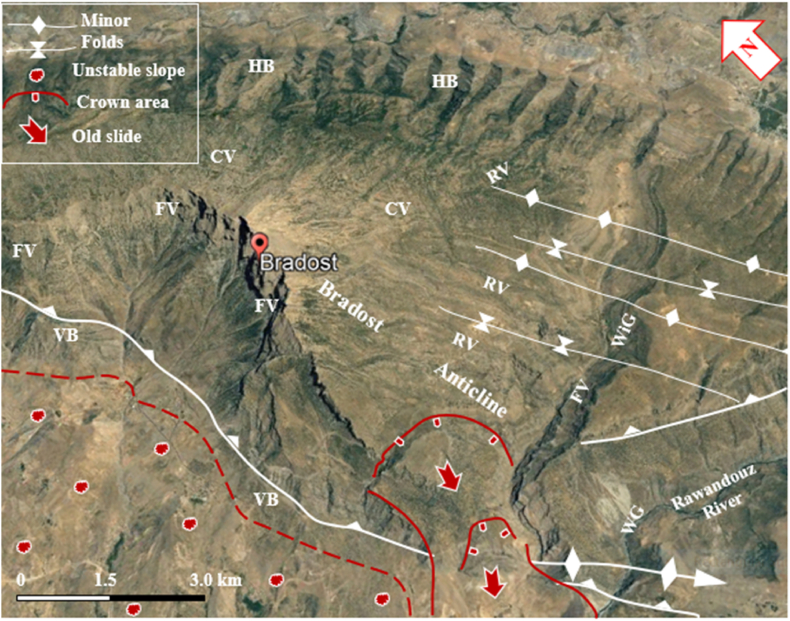
Satellite image of the Bradost anticline. Note the Hogbacks (HB), Fork-shaped valleys (FV), Cross-shaped valleys (CV), Water gap (WG), Wind gap (WiG), and vertical beds (VB).

**Fig. 8:**
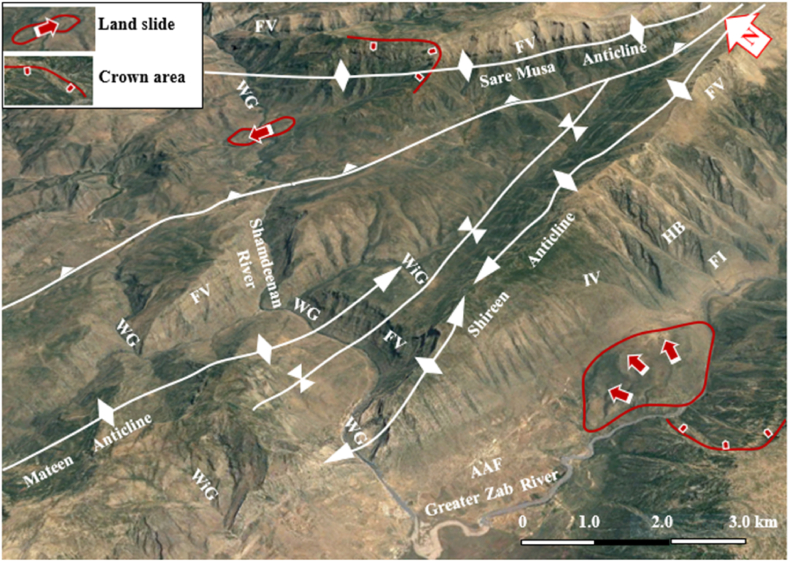
Satellite image of the Shireen and Sare Musa anticlines. Note the Hogbacks (HB), Fork-shaped valleys (FV), Water gap (WG), Wind gap (WiG), Abandoned alluvial fans (AAF), Landslides, and crown areas. Figure (1) shows the location of the image.

The studied four anticlines form a very rugged landform that is dissected by deep valleys; many of them form water and wind gaps. Steep cliffs are another characteristic form of the landscape. The highest elevated point among the four anticlines is on the top of the Sare Musa Mountain, it is 2333 m (a.s.l.), whereas the lowest point is at the merging point of the Rawandouz River with the Greater Zab River, which is about 402 m (a.s.l.) (Fig. 1). The studied anticlines are characterized by flat irons, Hogbacks (HB), and anticlinal ridges (AR) (Fig. 4), and intensive karstified areas (Fig. 5, Fig. 6 a). Different shapes of valleys are developed in the area; among them are: Fork-shaped (FV) (Fig. 4), inclined (IV) (Fig. 5), and radial (RV) (Fig. 6 b). The valleys flow in a perpendicular direction to the axes of the folds, which means parallel to the dip; however, locally they are not, like the inclined (Fig. 5, Fig. 8) and cross-shaped valleys (Fig. 7). Water gap (WG), wind gap (WIG), and wine glass forms (WGs) are very common in the studied area (Fig. 4, Fig. 5).

**Fig. 9:**
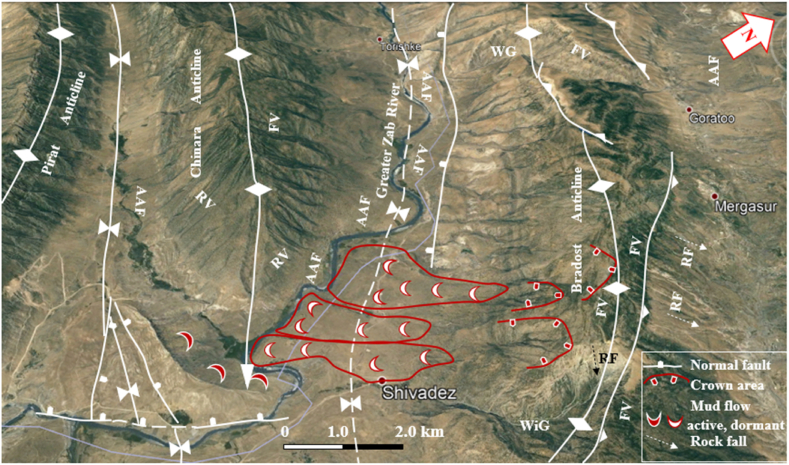
Satellite image of the Bradost and Chinara anticlines. Note the Abandoned alluvial fans (AAF), Radial valleys (RV), Fork-shaped valleys (FV), Water gaps (WG), Wind gaps (WiG), Rock fall (RF). For the location, refer to Fig. 1.

Hogbacks are developed locally; for example, within the rocks of the Shiranish Formation along the northeastern limb of the Bradost anticline (Fig. 7). Karstification is another characteristic feature of the carbonate rocks within the studied anticlines, like caves of different sizes and honeycomb carrens, especially in Shireen anticline (Fig. 6 a). Abandoned alluvial fans are developed along all four studied anticlines (Fig. 1, Fig. 2); usually at the outlets of some deeply incised valleys, which are not supplying the fans by sediments anymore due to the lateral growth of the anticlines and climatic change. The alluvial fans, which are abandoned now have shifted the Greater Zab River along its course during the development of the fans (Fig. 9). Landslides are also developed but are very old, some of them have changed the course of the Greater Zab River (Fig. 5), however, recent landslides can be seen at different parts of the studied anticlines.

For the location, refer to Fig. 1.

A very special case is recognized, where the slid mass has moved up to the southwestern limb of the Shireen anticline crossing the Greater Zab River, which flows now in the opposite direction of the slid mass (Fig. 8). Some rockfalls were recognized along the Bradost anticline (Fig. 9), and mud flows along the plunge of the hanging syncline (Fig. 9).

### 3.2 Tectonics and structural geology

The studied area is within the High Folded Zone (HFZ) which is part of the Zagros Fold – Thrust Belt and the HFZ covers the northeastern part of the Arabian Plate [28]. All anticlines have NW – SE trend with local swings of their axes, they range in length from (30–55) km (Table 1), whereas their widths range from (2.5–9.5) (Table 1).

The four folds are formed like those in the competent group of the Zagros Simply Folded Belt (HFZ in Iraq), they are formed by buckling and developed by a combination of flexural-slip and neutral-surface [29]. In the Zagros Range, including the HFZ, the maximum principal stress is normal to the fold axes and tangential to the earth's surface while the intermediate principal stress is parallel with the fold axes [29].

The four studied anticlines show many thrust faults (Fig. 5, Fig. 7, Fig. 8, Fig. 9), majority of the thrust faults are almost parallel to the fold axes leading to the disappearance of the fold axis; partly or totally. Locally, even the existing syncline between two anticlines has been covered by the thrust sheet like in the Shireen and Sare Musa anticlines (Fig. 5, Fig. 8). Back thrust faults also were recognized at the northwestern plunge area of the Chinara anticline (Fig. 4).

**Fig. 10:**
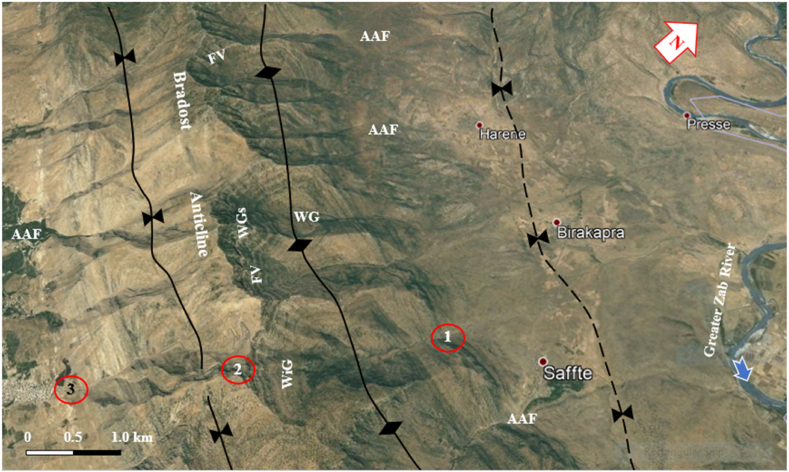
Satellite image of the Chinara anticline. Note the developed domes along the axes of the anticline and syncline. WG = water gap, WiG = Wind gap, WGs = Wine glass form, AAF = Abandoned alluvial fan, FV= Fork-shaped valley. Points 1, 2, and 3 refer to a very old wind gap. For the location, refer to Fig. 1.

**Fig. 11:**
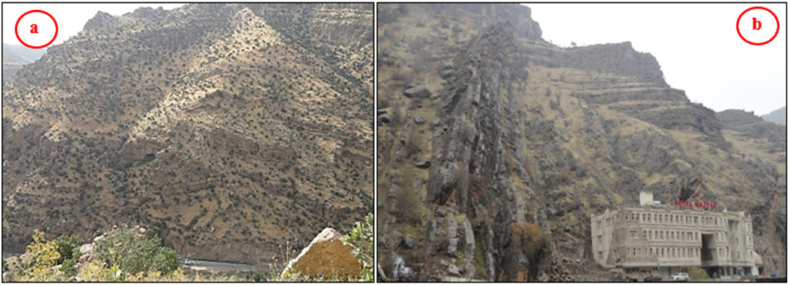
The Bradost anticline, a) Faulted beds in the southwestern limb,b) The southeastern plunge near Bekhal springs.

Normal faults also occur along the Bradost anticline and the plunge area of the existing syncline between the Chinara and Pirat anticlines (Fig. 9). Domes were recognized along the Chinara anticline (Fig. 10); not only in the axial part but even in the minor syncline that has developed along its southwestern limb (Fig. 10). Minor folds, which have developed along a limb of an anticline were also recognized along the southwestern limb of Chinara anticline (Fig. 4) and northeastern limb of Bradost anticline (Fig. 7). These developed minor folds gave the Bradost anticline an abnormal shape, seeming that the anticlinal axis extends along the northeastern limb. Many minor faults also occur along the four anticlines (Fig. 11), they are of different types with different displacements.

### 3.3 Stratigraphy

**Fig. 12:**
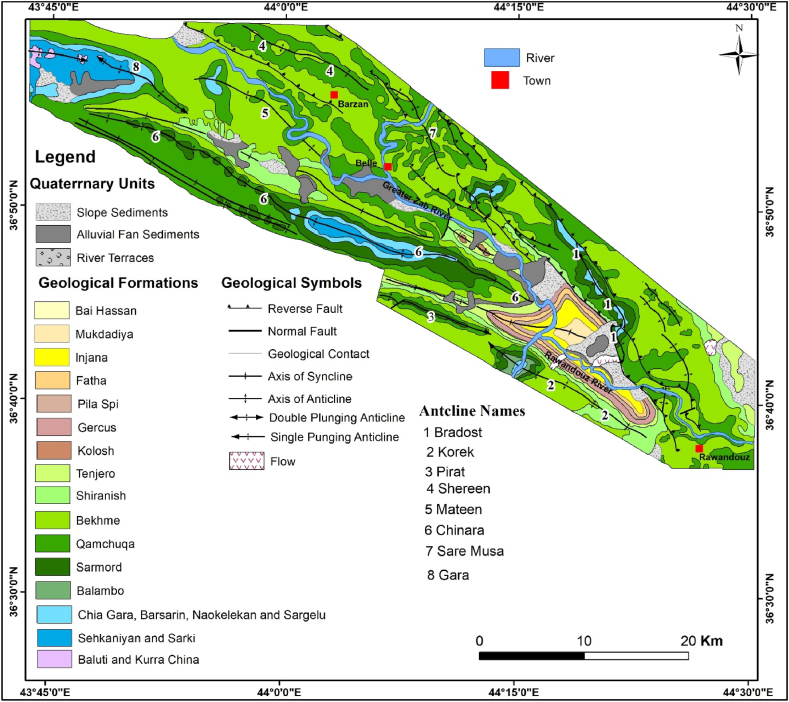
Geological map of the Korek and Pirat anticlines (modified and redrawn using GIS technique from Ref. [30]).

**Fig. 13:**
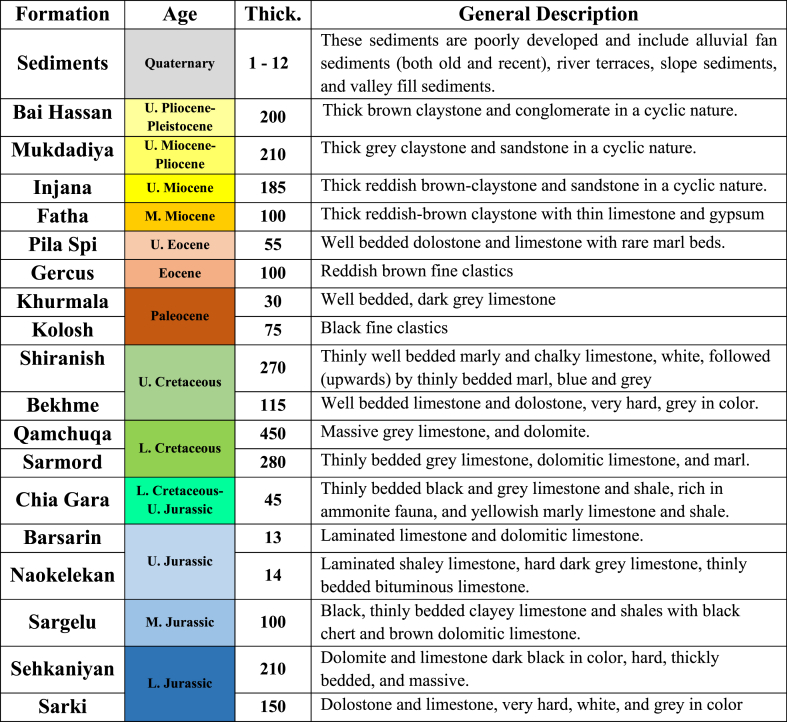
Generalized lithological columnar section of the exposed geological formations (Not to scale).

The exposed formations in the studied area are presented in the geological map (Fig. 12), whereas the general lithology of the exposed formations is briefed and shown in Fig. (13); based on Sissakian and Fouad [30].

## 4 Results

From the interpretation of satellite images, reviewing geological maps, and field investigation, we have acquired the following data about the four studied anticlines.

### 4.1 Bradost anticline

**Fig. 14:**
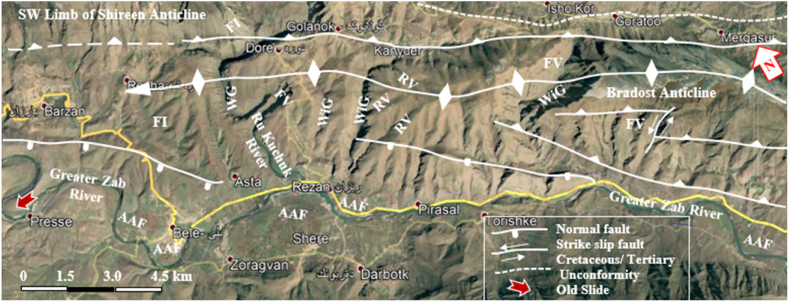
Satellite image of Bradost anticline, note the three types of faults. For the location, refer to Fig. 1.

The Bradost anticline is a double plunging anticline trending NW – SE with a length of 47.8 km and width of 9.39 km, it is worth mentioning that the true width can be measured only in one locality (Fig. 3, Fig. 7, Fig. 9, Fig. 12), this is attributed to reduced width due to existing thrust faults. The northwestern plunge is near the Ru Kuchuk stream whereas the southeastern plunge is near Bekhal springs (Fig. 11b), which forms a water gap (Fig. 12). There are three thrust faults along the anticline (Fig. 7, Fig. 14).

**Fig. 15:**
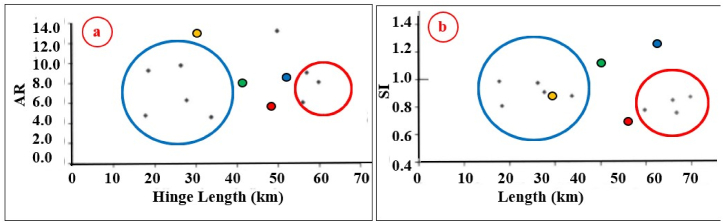
Indicators of fold types [24] **a)** AR, and **b)** SI. The black dots and the blue and red circles represent detachment and fault-bend folds, respectively [24]. The red, blue, green, and yellow dots represent Bradost, Chinara, Shireen, and Sare Musa anticlines

**Fig. 16:**
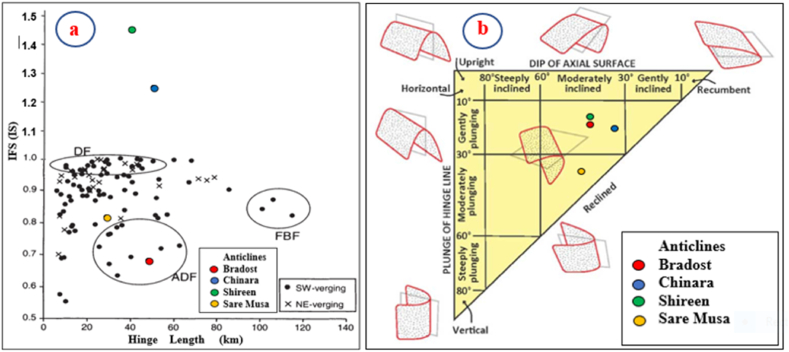
Three types of folds [24] a) IFS versus hinge length. DF = Detachment Fold, FBF = Fault-Bend Fold, and ADF = Asymmetric Detachment Fold [24], the four colored dots represent the four studied anticlines, and b) Classification of folds [13].

According to Burberry et al. [24], the Aspect Ratio (AR) and Fold Symmetry Index (IFS) or (SI) can be used to indicate the type of folding. The acquired AR and SI values of the Bradost anticline (Table 1) were imposed in Figure (15); accordingly, we found that the anticline is a “Detachment Fold”. We have checked the type of the detachment fold (Fig. 16a); accordingly, we found that the Bradost anticline is an Asymmetric Detachment Fold. The axial plane of the Bradost anticline shows a 33⁰ SW dip with an average 18⁰ dip of both plunges (Table 1), therefore the Bradost anticline is a moderately inclined fold with a gentle dipping plunge (Fig. 16b). The calculated value of the Smf of the Bradost anticline is 1.11 (Table 1). This index reflects the balance between erosion forces that tend to cut embayment into a mountain front and tectonic forces that tend to produce a straight mountain front coincident with an active range-bounding fault [31].

Different geomorphological forms were interpreted along the Bradost anticline and some of them were checked during field investigations, like: Water gaps, wind gaps, flat irons, abandoned alluvial fans, inclined valleys, radial valleys, cross-shaped valleys, fork-shaped valleys (Fig. 7, Fig. 9, Fig. 14), these features are excellent indications for the lateral growth of the anticline [[14], [15], [16], [17], [18]]. Moreover, old landslides, mudflows, and active rock fall were recognized (Fig. 9).

### 4.2 Chinara anticline

The Chinara anticline is a double plunging anticline with an NW – SE trend, a length of 53.1 km, and a width of 5.84 km (Table 1). The southeastern plunge is near the Greater Zab River, and it shows typical radial valleys (Fig. 9), and the northwestern plunge is covered by a thrust fault, however, it forms an en-echelon plunge with the Mateen anticline (Fig. 4). The Chinara anticline is multi domal (Fig. 12) all the domes are currently merged; its southwestern limb shows many minor folds (Fig. 4), and one long syncline (Fig. 12). The acquired AR and SI values of the Chinara anticline (Table 1) were applied in Figure (15); accordingly, we found that the anticline is near to “Fault Bend Fold”, but when we have checked the type of the detachment fold (Fig. 16a); accordingly, we found that the Chinara anticline is a Detachment Fold, this contradiction is discussed in the Discussion section (5.2). The axial plane of the Chinara anticline is dipping 33⁰ SW and the average dip of both plunges is 22⁰ (Table 1), therefore, the Chinara anticline is a moderately inclined fold with a gentle dipping plunge (Fig. 16b). The calculated value of the Smf of the Chinara anticline is 1.09 (Table 1).

Different geomorphological features were interpreted along the Chinara anticline and some of them were checked during field investigations, like: Water gaps, wind gaps, flat irons, abandoned alluvial fans, inclined valleys, radial valleys, fork-shaped valleys (Fig. 4, Fig. 9, Fig. 10), all these features are good indicators for the lateral growth of the anticline [[14], [15], [16], [17], [18]]. Moreover, an active mudflow was recognized near the southeastern plunge (Fig. 9).

### 4.3 Shireen anticline

The Shireen anticline is a double plunging anticline trending NW – SE with a length of 37.7 km and a width of 5.46 km (Table 1). The southeastern plunge is near the Rawandouz River, it exhibits a very old landslide (Fig. 5), whereas the northwestern plunge is near the Shamdeenan River forming a water gap (Fig. 5). The Shireen anticline shows one small dome near its northwestern plunge (Fig. 5), parts of its northeastern limb is thrusted over the southwestern limb causing the disappearance of the axial part of the anticline (Fig. 5). The acquired AR and SI values of the Shireen anticline (Table 1) were applied in Figure (15); accordingly, we found that the anticline is closer to the “Detachment Fold”. When we applied the opinion of Burberry et al. [24]; however, we found it is a clear Detachment Fold (Fig. 16 a). The axial plane of the Shireen anticline is dipping 41⁰ NE and the average dip of both plunges is 16⁰ (Table 1), therefore the Shireen anticline is a moderately inclined fold with gentle dipping plunge (Fig. 16 b). The value of the Smf of the Shireen anticline is 1.04 (Table 1).

Different geomorphological features were interpreted along the Shireen anticline and some of them were confirmed during field investigations, among them are: Water gaps, wine glass form, hogbacks, inclined valleys, and fork-shaped valleys (Fig. 5, Fig. 8), these features are excellent indications for the lateral growth of the anticline [[14], [15], [16], [17], [18]]. Moreover, a very intensively karstified area was recognized on the top of the anticline (Fig. 5, Fig. 6a), and many old landsides, which shifted the course of the Greater Zab and Rawandouz rivers (Fig. 5).

### 4.4 Sare Musa anticline

The Sare Musa anticline was not named in all existing geological maps; therefore, we used this name based on the highest peak's name on the top of the anticline. The Sare Musa anticline is a double plunging anticline with an NW – SE trend, a length of 32.2 km, and a width of 3.24 km (Table 1). The southeastern and northwestern plunges clear; however, part of the axial part is hindered by a large and old landslide (Fig. 5, Fig. 8). The acquired AR and SI values of the Sare Musa anticline (Table 1) were applied in Figure (15); accordingly, we found that the anticline is a “Detachment Fold”. When we applied the opinion of Burberry et al. [24]; to check the type of the Detachment Fold (Fig. 16 a), it is clearly shown that it is an Asymmetrical Detachment Fold (Fig. 16 a). The axial plane of the Sare Musa anticline is dipping 43⁰ NE and the average dip of both plunges is 38⁰ (Table 1), therefore the Sare Musa anticline is a moderately inclined fold with a moderately dipping plunge (Fig. 16b). The calculated value of the Smf of the Sare Musa anticline is 1.30 (Table 1).

Different geomorphological features were interpreted along the Sare Musa anticline, among them are: Water gaps, and fork-shaped valleys (Fig. 5, Fig. 8), these features are excellent indications for the lateral growth of the anticline [[14], [15], [16], [17], [18]]. Moreover, a very intensively karstified area was recognized on the top of the anticline (Fig. 5), and many old landsides, which have shifted the course of the Shamdeenan River (Fig. 8), and well-developed hogbacks and flat irons (Fig. 8).

## 5 Discussion

The Bradost, Chinara, Shireen, and Sare Musa anticlines were studied using satellite images to interpret and deduce the structural and geomorphological characteristics, some of the interpreted forms (which are accessible) were checked in the field. Accordingly, we have found a lot of data that were not shown in the geological map (Fig. 12). This is attributed to: 1) the scale of the present geological maps, 2) the scale of the carried-out work as compared to the present work, and 3) the quality of the used satellite images.

The four studied anticlines are Detachment Folds (Fig. 15, Fig. 16a), as the symmetry index allows the folds to be clearly divided into two end-member groups, fault-bend folds, and detachment folds [24]. According to Burberry et al. [24], the Detachment folds are seen to cluster with aspect ratios of less than 10 and hinge lengths of less than 60 km, and can also be seen to have a symmetry close to unity. In the four studied anticlines, the recorded aspect ratios, hinge lengths, and symmetry index show that they all are Detachment folds (Table 1).

The four studied anticlines exhibit thrust faults (Fig. 4, Fig. 5, Fig. 7, Fig. 12, Fig. 14), which have changed their geometry and shape. Therefore, the recorded values of the aspect ratio and symmetry index are not common (Table 1 and Fig. 15, Fig. 16a), since “Detachment folds have a low aspect ratio and near-perfect symmetry (Suppe 1983 in Ref. [24])”. This is attributed to intensive thrust faults, which have dissected the anticlines and locally hindered their true lengths and widths (Fig. 4, Fig. 5, Fig. 8, Fig. 14), and it is also well known that in young fold-thrust belts such as the Zagros, the geometry is reflected topographically by concordant landform morphology [24].

**Fig. 17:**
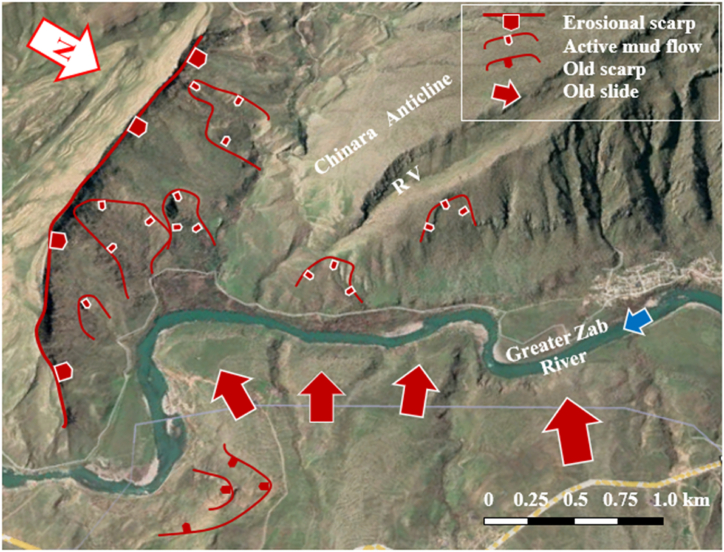
Active mud flows in the plunge of the Chinara anticline, and old landslides along the Bradost anticline. For the location, refer to Fig. 1.

The presence of water gaps, wind gaps, differently shaped valleys, and domes are all good indications that the four studied anticlines are exhibiting lateral growth [[14], [15], [16], [17], [18]]. Abandoned alluvial fans (Fig. 17) are also good indications of active folds and their lateral growth [32]. Along the four studied anticlines, many abandoned alluvial fans were recognized (Fig. 8, Fig. 9, Fig. 10, Fig. 14). Some of them have shifted the courses of the Greater Zab, Rawandouz, and Ru Kuchuk rivers, indicating they are very old fans (Fig. 8, Fig. 9, Fig. 14). Moreover, the water gaps indicate that the fold uplift rate is slow relative to the stream incision rate. Whereas wind gaps are caused by the abandonment of the stream channel where the uplift rate is higher; that is, nearer the central point of a detachment fold. Such clear cases occur in the Bradost anticline (Fig. 7, Fig. 14), and the Chinara anticline (Fig. 10), where the trace of a wind gap is very clear although on top of the anticline (Points 1, 2, and 3 in Fig. 10), in the Shireen anticline (Fig. 8), and in the Sare Musa anticline (Fig. 5). The presence of wind gaps at the crests of folds is also a good indication that they are detachment folds [24].

According to Sepehr and Cosgrove [33], the geometry of many long linear anticlines with a high aspect ratio along the Zagros Frontal Fault implies thrust involvement. The Chinara anticline is a good example in the studied area since its hinge length, length, and Mountain Front Sinuosity index (Smf) indicate that it is a long linear anticline with a high aspect ratio (Table 1).

Moreover, the sinuosity index (Smf) can be used to indicate the relative ages of folds, a higher sinuosity measurement implies a heavily incised fold front and thus an older fold structure (Azor et al., 2002 and Silva et al., 2003 in Ref. [24]). The recorded Smf values are low (Table 1); therefore, they indicate that the age of the folding is young.

Landslides were recognized at different parts of the four studied anticlines (Fig. 5, Fig. 7, Fig. 8, Fig. 9, Fig. 14, Fig. 17). Along the Bradost anticline, several very old landslides were recognized (Fig. 9). In some of them, the reactivated slide was recognized within the main old slide mass. Along the southwestern limb of the Shireen anticline, a large slide mass was recognized, it was slid from the Mateen anticline and crossed the Greater Zab River, as indicated from the crown area on the northeastern limb of the Mateen anticline (Fig. 8). Recent mud flows were recognized between the southeastern plunge of the Chinara anticline and the neighboring plunging syncline (Fig. 9, Fig. 17). Some of the recent and active mud flows are about (150–300) m in width and (100–600) m in length, they are caused by the lateral erosion and undercut of the slopes by the Greater Zab River (Fig. 17). Some active landslides can be seen on the plunge and northeastern limb of the Chinara anticline (Fig. 17). One of the radial valleys on the southeastern plunge of the Chinara anticline is enlarged; due to extensive erosion forming a grove like a valley (Fig. 17). Some of the mentioned geomorphic forms were used by different researchers [[34], [35], [36]] to indicate active faults and Neotectonic activities.

Other geomorphological forms which were recognized are hogbacks and whaleback anticline, like in a part of the Bradost anticline (Fig. 7, Fig. 8), flat irons in all anticlines, and rounded tip folds; like the Chinara anticline (Fig. 9, Fig. 17). Such rounded tip folds are a good indication of the presence of thick, low-friction ductile detachment below such folds [37]. The presence of whaleback anticlines is a good indication that the decollement is shallow (Sepehr and Cosgrove, 2007 in Ref. [24]). In the studied area, the decollement rocks are most probably the Triassic age rocks of the Baluti Formation (black shale) and Kura Chine Formation (Dolomite).

The values of the Smf index are divided into three classes: Class 1) High, Smf = 1.0 to 1.5, Class 2) Moderate, Smf = 1.5 to 2.5, and Class 3) Low, Smf > 2.5 [38]. Therefore, the Chinara and Shireen anticlines belong to Class 1, whereas the Bradost and Sare Musa anticlines belong to Class 2 (Table 1). Accordingly, the Chinara and Shireen anticlines show Hight tectonic activity, whereas the Bradost and Sare Musa anticlines show Moderate tectonic activity.

## 6 Conclusions

The studied Bradost, Chinara, Shireen and Sare Musa anticlines are Detachment folds; as indicated by their measured aspect ratio, fold symmetry index, hinge length, length, and width of the anticlines. The measured Smf values indicate that the Chinara and Shireen anticlines exhibit High tectonic activity, whereas the Bradost and Sare Musa anticlines exhibit Moderate tectonic activity. The recognized geomorphological forms (water and wind gaps, differently shaped valleys, and abandoned alluvial fans) and structural forms (domes and en-echelon plunges) indicate that all four studied anticlines are laterally growing, and the stream incision rates are larger than the fold growth rates. Different mass movement phenomena have recognized majority are very old; however, recent, and active mud flows were recognized in the southeastern plunge area of the Chinara anticline. Other phenomena are old landslides, rock fall, and mud flow. Severely karstified large areas were recognized on the top of Shireen and Sare Musa anticlines.

## Author contribution statement

Varoujan K. Sissakian; Ala A. Gahfur; Nadhir Al-Ansari: Conceived and designed the experiments; Performed the experiments; Analyzed and interpreted the data; Contributed reagents, materials, analysis tools or data; Wrote the paper.

Hassan O. Omer; Hawkar A. Abdulhaq: Conceived and designed the experiments; Analyzed and interpreted the data; Contributed reagents, materials, analysis tools or data.

## Funding statement

Apart from the provided logistics by 10.13039/501100007781UKH to perform the field work, no other fund was received by the authors to perform this research work.

## Data availability statement

Data will be made available on request.

## Declaration of competing interest

The authors declare that they have no known competing financial interests or personal relationships that could have appeared to influence the work reported in this paper.
